# Development and conduction of an active re-implementation of the Norwegian musculoskeletal guidelines

**DOI:** 10.1186/s13104-018-3894-4

**Published:** 2018-11-03

**Authors:** Ann Mari Gransjøen, Siri Wiig, Kristin Bakke Lysdahl, Bjørn Morten Hofmann

**Affiliations:** 1 Department of Health Sciences in Gjøvik, Norwegian University of Science and Technology in Gjøvik (NTNU), Teknologiveien 22, 2815 Gjøvik, Norway; 2 Faculty of Health Sciences, SHARE-Centre for Resilience in Healthcare, University of Stavanger, Kjell Arholmsgate 41, 4036 Stavanger, Norway; 3 Department of Optometry, Radiography and Lighting Design, Faculty of Health Sciences, University of South-Eastern Norway, PO Box 235, 3603 Kongsberg, Norway; 4 Center for Medical Ethics, University of Oslo, PO Box 1130, Blindern, 0318 Oslo, Norway

**Keywords:** Guideline implementation, Intervention development, Primary care, Radiology, Musculoskeletal, Non-traumatic

## Abstract

**Objective:**

Significant geographical variations in the use of diagnostic imaging have been demonstrated in Norway and elsewhere. Non-traumatic musculoskeletal conditions is one area where this has been demonstrated. A national musculoskeletal guideline was implemented in response by online publishing and postal dissemination in Norway in 2014 by national policy makers. The objective of our study was to develop and conduct an intervention as an active re-implementation of this guideline in one Norwegian county to investigate and facilitate guideline adherence. The development and implementation process is reported here, to facilitate understanding of the future evaluation results of this study.

**Results:**

The consolidated framework for implementation research guided the intervention development and implementation. The implementation development was also based on earlier reported success factors in combination with interviews with general practitioners and radiologists regarding facilitators and barriers to guideline adherence. A combined implementation strategy was developed, including educational meetings, shortening of the guideline and easier access. All the aspects of the implementation strategy were adapted towards general practitioners, radiological personnel and the Norwegian Labor and Welfare Administration. Sixteen educational meetings were held, and six educational videos were made for those unable to attend, or where meetings could not be held.

**Electronic supplementary material:**

The online version of this article (10.1186/s13104-018-3894-4) contains supplementary material, which is available to authorized users.

## Introduction

Radiology has been an important part of diagnostics for a long time, and as the field has become more advanced and complex, the last decades have seen an increased use of diagnostic imaging services [1, 2]. This has led to an increased need for guidelines; thus the American College of Radiologists and the Royal College of Radiologists, among others, developed referral guidelines in the early 90s for diagnostic imaging. These guidelines are decision aids for referrers, who give recommendations regarding the optimal examination(s) for a specific clinical question [3]. Sufficient resources for maintaining national guidelines broadly covering all clinical questions have not been allocated in Norway.

Significant geographical variations in the use of diagnostic imaging have been demonstrated in Norway, as elsewhere, and waiting times have increased [4]. Approximately 19% of all consultations in primary care were found to be about complaints from the musculoskeletal system, and a large portion of these patients would be referred to diagnostic imaging [4]. The Norwegian Directorate of Health developed the national musculoskeletal guideline for these reasons, and implemented it by online publishing and postal dissemination in 2014. This guideline provides recommendations regarding the use of the four most commonly used modalities, conventional radiography (CR), computed tomography (CT), magnetic resonance imaging (MRI) and ultrasound (US) for the diagnosis of non-traumatic musculoskeletal diseases [4].

The available guidelines appear to be sparsely known in primary care and the radiological field, and a minority of general practitioners, who are the main target group for the guideline, report using them (2/8 interviewed GPs reported actively using them) [5].

Several methods of implementing the guidelines regarding diagnostic imaging have been tried, but the effects on radiological practice is quite variable [6–9]. It has been seen, however, that the more educational implementation strategies (seminars, reminders, feedback) [10–13] and strategies delivered during the decision moment (reminders, posters and cards used during patient consultation) [14, 15] the greater their effect is than postal dissemination, for example. This has also been found in other areas of medicine [16]. Yet, even if an effect is observed immediately after an implementation, it may not last over time [17]. However, it has been found that education about, and easy access to, the guideline, changes in the referral form, and combinations of different methods have a more positive effect on changing practice behavior over time [18, 19].

This study’s objective was to develop and conduct an active re-implementation of the Norwegian musculoskeletal guideline to investigate and facilitate guideline adherence. This is important, since this guideline is not used extensively in primary care, and few radiological personnel know of its existence [5]. The aims of the re-implementation were to facilitate adherence to the Norwegian musculoskeletal guideline and to potentially increase the appropriateness of the diagnostic imaging applied to non-traumatic musculoskeletal diseases.

We report here on the development and implementation process as additional data provided to facilitate understanding of the future evaluation results of this study.

## Main text

### Development of the re-implementation

#### Setting and population

The intervention was carried out in one county in Norway, and the primary target groups were practicing general practitioners (GPs) and radiologists. GPs were chosen since the guideline is aimed at primary health care providers; however, radiologists will also benefit from knowledge of the guideline. Radiologists are obliged by law to ensure that unjustified examinations are changed or stopped; thus they were also included as a target group, and because they extensively collaborate with radiographers. The radiographer is often the first, and only, person to assess the referral to CR examinations.

The Norwegian Welfare and Labor Administration (NAV) were also included because of their collaboration with GPs. NAV will benefit from knowledge of the guideline to avoid requiring GPs to make referrals to diagnostic imaging, as this may not be justified in some cases, but is wanted to document disease.

#### Recruitment

First author AMG made recruitment through telephone or e-mail contact with the different medical offices and medical centers and gave information about the interventions content and process. They decided whether to participate or not on the basis of the information. Telephonic contact was made when an educational meeting could not be held to inform the potential participants about the intervention, and their e-mail addresses were collected to enable circulation of links to educational videos. Information was also provided about a shortened versioned of the guideline that would be sent through the regular mail.

#### Development of the implementation strategy

The project’s first phase comprised interviews with GPs and radiologists to find barriers and facilitators for guideline adherence [5]. Information on facilitators and barriers found in the interviews was used to decide on the components of the implementation strategy, supplemented by the implementation literature were previously successful, to determine if any of these could be used in addition to the information from the interviews.

The main points raised in both the interviews, and in the literature, were lack of education regarding the guidelines (lack of familiarity), the guidelines being too long, and a need for easier access [5, 20, 21]. This resulted in the development of a combined strategy with educational meetings, shortening of the guideline through extractions of the already existing recommendations and providing easy access to the guideline.

**Fig. 1:**
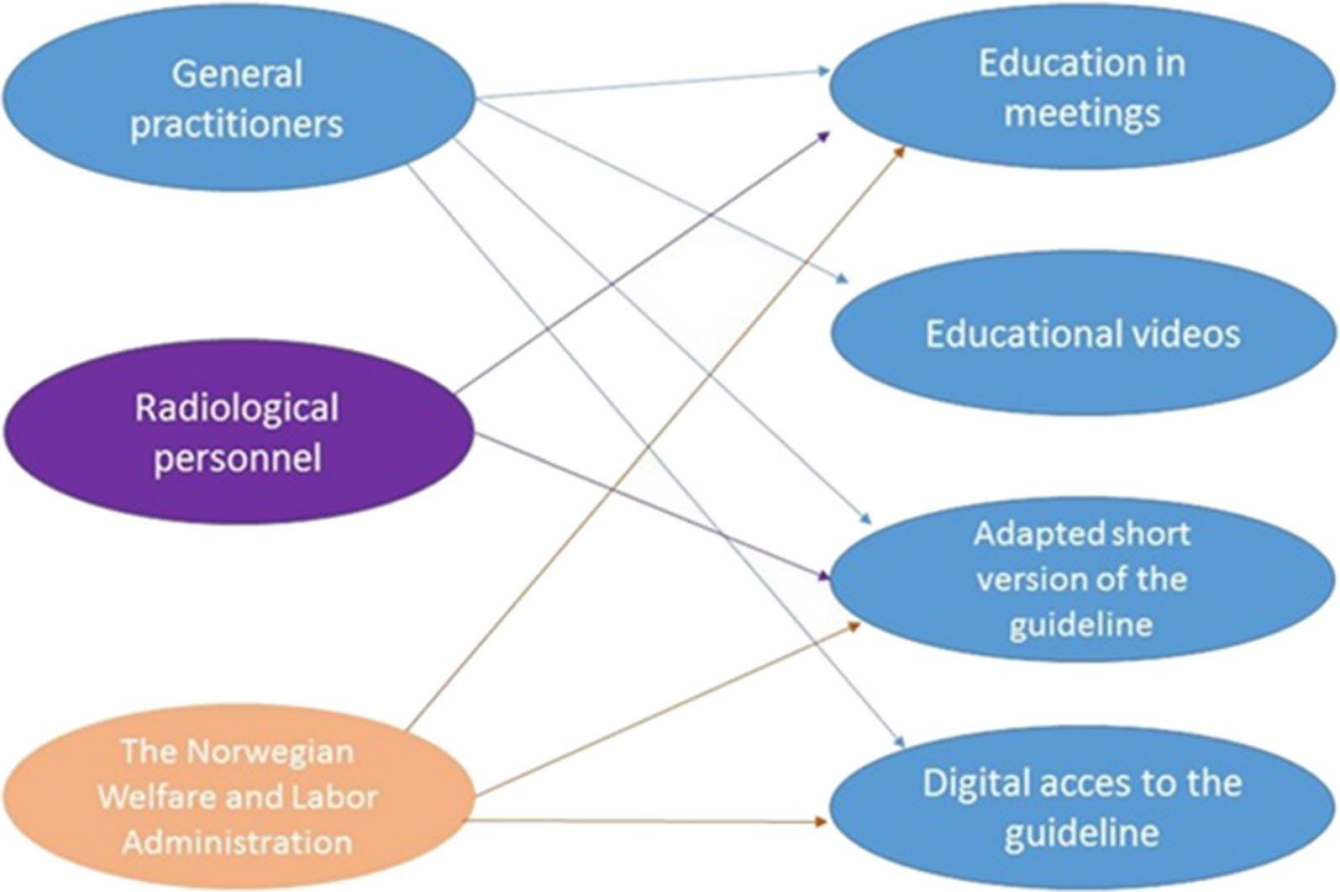
Overview of the intervention content for the included intervention groups

The process construct from the consolidated framework for implementation research (CFIR) was also used as a guide, as it explains elements that could be important for the implementation (for example, the use of champions and opinion leaders), and underline the importance of planning and executing the intervention to plan [22]. See Fig. 1 for a complete overview of the intervention, including its different parts that the groups received during the intervention.

Parts of the educational element and the shortened guideline were developed in cooperation with a practice coordinator as a liaison between the primary and secondary care practitioners, because of the coordinators’ experience in primary care. This included an overview of the cooperation between primary care and secondary care, thereby functioning as a sort of champion in this case. Since the most common musculoskeletal complaints from patients seen in primary care facilities in Norway are regarding the neck, shoulders, lower back and knees [23], the focus for the shortened guideline and the educational component was on these areas. This was done by making an excerpt of the pre-existing short version of the recommendations published by the Norwegian Directorate of Health into a flow-sheet on a laminated sheet of A4-paper, which could easily be kept at the users’ desks. The guideline was also published in the Norwegian clinical manual (NEL), a general medical encyclopedia aimed at health professionals, and the most used digital resource by the GPs, to further facilitate its accessibility.

The short version was also adapted to the radiological departments, so it could be kept at the workstations or x-ray labs. The same style and format was chosen for the radiological departments as for the GPs, however, the information presented was somewhat different. The general information about the modalities was removed, as it was perceived as unnecessary. Radiological personnel had no digital resource corresponding to NEL and were, therefore, provided with links to two other existing online resources for the guideline (the Health Directorate’s web page and the Norwegian digital health library). The educational component was also adapted towards the radiological personnel.

### Conducting the re-implementation

The intervention’s educational component was an educational meeting conducted by AMG, who has first-hand knowledge and experience from the field as a trained radiographer. The educational meetings consisted of information about the guideline, as well as questions and discussions about the guideline. Their focus was on the recommendations given regarding the optimal examinations for patients with pain from the neck, shoulder, lower back or knee, as well as the guideline’s aims and rationale. There was also a focus on why this guideline could be relevant for the radiological personnel’s work and how it could be used in their workplace.

These educational meetings were held in several arenas, such as in-house trainings, morning or lunch meetings with single medical centers, or larger meetings that included all the medical practices in a municipality. Each meeting lasted between 30 and 60 min, depending on the number of attendees. The larger meetings usually lasted longer because of more discussions around the table. The benefit of holding these meetings in pre-existing arenas was that the attendees did not have to take extra time out of their schedule, making it more likely for them to attend. Table 1 provides an overview of the participants in the educational meetings.

**Table 1:** Overview of the participants from the different target groups and sites visited in the educational meetings

Target group	No./total number	Percentage of total	Sites visited
GPs	113/213	53%	12
Radiological personnel	28	N/A	3
NAV	5/7	71%	1

Meetings were held with 12 of the county’s 26 municipalities between November 2017 and February 2018; given the meetings’ attendance, approximately 59% of the counties’ GPs received the education through the meetings. Educational videos were made for the remaining 14 municipalities and the GPs not attending the meetings. Six videos were developed that were equivalent to the education given during the meetings, presented by the first author. See Additional file 1 for details regarding the development and contents of the videos.

These educational meetings were also adapted for, and held at all three of the county’s radiological departments (two public hospitals and one private institution). The educational content was adapted towards radiological personnel; as previously mentioned, it was decided to include both radiographers and radiologists because radiographers are often the first, and only, to assess the referral. Those meetings lasted approximately 30 min, and the majority were held as lunch-meetings. The education for this group also covered an introduction to the guidelines and the recommendations regarding patients with pain from neck, shoulder, lower back and knee. How radiological personnel could use this guideline and why it could be beneficial in their day-to-day work was the focus in order to facilitate guideline adherence, since this guideline is not primarily aimed at them. The general information of the different modalities was not included, since this was perceived as unnecessary for this target group.

A 45 min meeting with NAV was also held in the same county; it covered the same topics as the meetings held with the GPs, as they included GPs and other specialties, and most would benefit from the same education as the GPs in the municipalities.

#### Evaluation

The intervention will be evaluated by quantitative measurements (such as a time series) of radiological procedures performed in two counties (the intervention county and one control), and by qualitative focus group interviews in the intervention county. These results will be published after the final measurements of effect are conducted in 2019.

## Limitations

A broader approach that included more counties and all relevant health-care professionals, thereby including chiropractors and manual therapists, could yield different results regarding the effects of, and challenges in, implementing the intervention, as well as improving the intervention’s transferability. However, this is an issue of resources, and major stakeholders have been included, in addition to a control county not receiving any intervention components.

It can also be seen as a limitation that the radiologists only had the educational meetings and shortened versions of the guidelines and did not receive adapted educational videos or a digital resource adapted for them. Another drawback is that NAV did not receive the educational videos. However, nearly all of its relevant personnel attended the meeting. Another limitation was that no validation study was performed on the educational videos or shortforms. However, they were direct extracts from the original guideline, and should therefore still be fairly accurate.

An additional limitation is that participation was voluntary, so all participants were tentatively positive about the guideline, wished to learn more about it and were motivated to increase their use of it. The implementation process may have been different if the participants had a more skeptical viewpoint.

## Additional file

**Additional file 1.** Development of the educational videos. Description of the development process of the educational videos used in the re-implementation of the Norwegian Musculoskeletal Guidelines.

## Abbreviations

CFIR: consolidated framework for implementation research
CR: conventional radiography
CT: computed tomography
GP: general practitioner
MRI: magnetic resonance imaging
NAV: Norwegian Labour and Welfare Services
NEL: Norwegian clinical manual
NSD: Norwegian Social Science Data Services
US: ultrasound
