# Supplementary material for: Development and conduction of an active re-implementation of the Norwegian musculoskeletal guidelines
Source: BMC Res Notes. 2018 Nov 3;11:785. doi: 10.1186/s13104-018-3894-4 (PMC6215611; doi:10.1186/s13104-018-3894-4)
Supplement: Supplementary file 1 — Additional file 1. Development of the educational videos. Description of the development process of the educational videos used in the re-implementation of the Norwegian Musculoskeletal Guidelines. [file 13104_2018_3894_MOESM1_ESM.docx]

**Additional file 1**

**Development of the educational videos.**

Filming was done using swivl (https://www.swivl.com/) in a studio at the first authors workplace. The recordings can later be accessed and shared through cloud storage. The videos were shared using links that were distributed through e-mails to the visited medical centers.

The contents of the videos were equal to the content of the educational meetings. There were six videos made in total, to reduce the length of the videos. The reduction in video length were done to facilitate viewing, as well as giving the option to view only the parts of the material seen as relevant for the individual viewer.

The first video consisted of the first author providing an introduction to the guidelines and the rationale that was used for developing and publishing the guidelines. Videos two through five consisted of the first author explaining the recommendations the guidelines provide for diagnostic imaging of non-traumatic diseases in the neck, shoulder, lower back and knee. The last video consisted of the first author giving two questions for thought, as well as further information regarding where the full version of the guidelines could be found online.
